# Plasma membrane association facilitates conformational changes in the Marburg virus protein VP40 dimer†
†Electronic supplementary information (ESI) available: Figures for rmsf and lipid protein interactions, movies showing membrane association and conformational changes in the dimer, and tables with information about simulation setup as well as hydrogen bond pairs. See DOI: 10.1039/c7ra02940c



**DOI:** 10.1039/c7ra02940c

**Published:** 2017-04-26

**Authors:** Nisha Bhattarai, Jeevan B. GC, Bernard S. Gerstman, Robert V. Stahelin, Prem P. Chapagain

**Affiliations:** a Department of Physics, Florida International University, Miami, FL 33199, USA. Email: chapagap@fiu.edu; b Biomolecular Sciences Institute, Florida International University, Miami, FL 33199, USA; c Department of Chemistry and Biochemistry, The Eck Institute for Global Health, The Boler-Parseghian Center for Rare and Neglected Diseases, University of Notre Dame, Notre Dame, IN 46556, USA; d Department of Biochemistry and Molecular Biology, Indiana University School of Medicine-South Bend, South Bend, IN 46617, USA

## Abstract

Filovirus infections cause hemorrhagic fever in humans and non-human primates that often results in high fatality rates. The Marburg virus is a lipid-enveloped virus from the Filoviridae family and is closely related to the Ebola virus. The viral matrix layer underneath the lipid envelope is formed by the matrix protein VP40 (VP40), which is also involved in other functions during the viral life-cycle. As in the Ebola virus VP40 (eVP40), the recently determined X-ray crystal structure of the Marburg virus VP40 (mVP40) features loops containing cationic residues that form a lipid binding basic patch. However, the mVP40 basic patch is significantly flatter with a more extended surface than in eVP40, suggesting the possibility of differences in the plasma membrane interactions and phospholipid specificity between the VP40 dimers. In this paper, we report on molecular dynamics simulations that investigate the roles of various residues and lipid types in PM association as well as the conformational changes of the mVP40 dimer facilitated by membrane association. We compared the structural changes of the mVP40 dimer with the mVP40 dimer in both lipid free and membrane associated conditions. Despite the significant structural differences in the crystal structure, the Marburg VP40 dimer is found to adopt a configuration very similar to the Ebola VP40 dimer after associating with the membrane. This conformational rearrangement upon lipid binding allows Marburg VP40 to localize and stabilize at the membrane surface in a manner similar to the Ebola VP40 dimer. Consideration of the structural information in its lipid-interacting condition may be important in targeting mVP40 for novel drugs to inhibit viral budding from the plasma membrane.

## Abstract 2

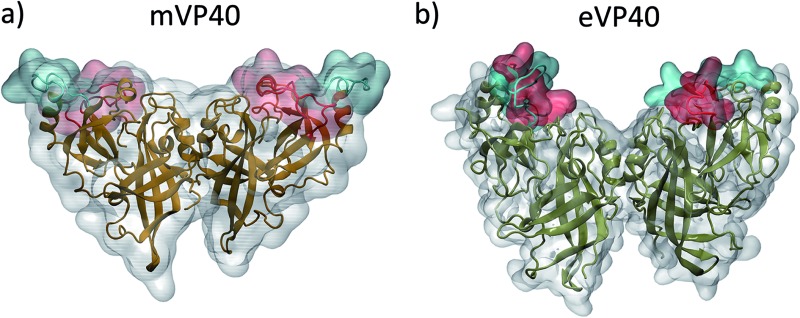
The membrane binding interface of the Marburg virus protein mVP40 dimer differs from that of the Ebola virus eVP40 dimer but membrane binding allows conformational changes in mVP40 that makes it structurally similar to the eVP40 dimer.

## Introduction

Filovirus infections cause hemorrhagic fever in humans and non-human primates^1^ that often results in high fatality rates.^2^ The Marburg virus (MARV) is a lipid-enveloped virus from the Filoviridae family and is closely related to the Ebola virus (EBOV). The virus particle acquires a lipid coat from the plasma membrane (PM) of the host cell as the virus assembles and buds.^3^ The viral matrix layer underneath the lipid envelope is formed by the matrix protein VP40, and provides shape and stability to the viral particle. It has been shown in mammalian cells that only the expression of the VP40 protein, among the EBOV and MARV genome, is sufficient for forming innocuous but authentic-looking virus-like particles (VLPs).^4^ VP40 is involved in multiple functions during the viral life-cycle^5^ and protein multifunctionality often requires proteins to undergo conformational changes.^6–8^ It has been shown that, depending on the function, EBOV VP40 (eVP40) exists in different conformations such as a butterfly shaped dimer involved in the transport of the protein to the membrane, a hexamer to form the viral matrix beneath the lipid-envelope, and an octamer ring structure to bind RNA and regulate viral transcription.^5^ The X-ray crystal structure determination of the dimeric, hexameric and octameric forms of eVP40 has provided a great deal of information about the structural transformation of the protein into various oligomeric states for performing different functions.^5^ Structural information of such viral proteins also offers an opportunity to explore potential inhibitors for treating the disease.^9^

Many proteins are known to interact with lipids in a membrane, and lipid binding can induce protein conformational changes that modulate their functions.^10–15^ In addition to the direct role of the protein–phospholipid interactions on the conformational changes and allosteric modulations of integral membrane proteins such as ion channels and receptors,^16–20^ lipid interactions at the membrane surface can induce structural changes in peripheral proteins.^21,22^ Depending on their various functional needs, some of these proteins interconvert between solution form and lipid-bound structure.^23,24^ A widely studied example is the human apolipoprotein E3, whose NTD has been shown to adopt an elongated globular four helix bundle structure in solution but undergo conformational changes upon lipid binding.^25^ It has been hypothesized that, upon membrane association, the filovirus VP40 dimers undergo major structural rearrangements. This step is required for oligomerization into hexameric structures^5,26^ which further assemble to form filaments leading to the formation of the viral matrix.^27^ However, the mechanisms and consequences of the VP40-lipid interactions on VP40 dynamics and assembly are not well understood in either eVP40 or mVP40.

The crystal structure of the MARV VP40 (mVP40) dimer has recently been determined.^28^ As in eVP40, the structure of the mVP40 dimer features an α-helical dimer interface in the N-terminal domains (NTD) as well as a basic patch in the C-terminal domain (CTD) that mediates membrane binding.^28^ It was found that mutations in the basic patch residues greatly hindered the mVP40 assembly and adversely affected VLP budding due to the reduction in anionic lipid binding caused by these mutations.^28^ This shows that the mVP40 dimer, like eVP40, is involved in trafficking of the protein to the lower leaflet of the PM and membrane localization *via* the basic patch. Structural comparison shows that the NTD structures as well as the NTD–NTD interfaces in both mVP40 and eVP40 dimers are quite similar. Given the large sequence similarity (42%) between the NTDs of mVP40 and eVP40, this is not surprising. In contrast, the mVP40 CTD is significantly different from the eVP40 CTD, with only a 15% sequence similarity. The crystal structure shows that the mVP40 CTD basic patch is significantly flatter with a more extended surface than that of eVP40.^28^ This suggests that the mVP40 dimer could interact with the PM differently than the eVP40 dimer, leading to differences in the phospholipid specificity, oligomerization and budding of the VLPs.^5,27,29–31^

Recently, Wijesinghe *et al.* used Hydrogen Deuterium Exchange Mass Spectroscopy to investigate at various timescales, structural changes in mVP40 due to phospholipid interactions.^32^ By determining the solvent accessibility of the mVP40 residues, important residues involved in binding to the membrane, as well as those residues at the oligomerization interface were identified. However, the structural details of the mVP40 conformation after membrane association is still lacking. In this paper, we investigated the PM association and the conformational changes of the mVP40 dimer induced by membrane association during the early stages of oligomerization at the plasma membrane. We compared the structural changes of the mVP40 dimer with the eVP40 dimer in both lipid free and membrane associated conditions. Despite the significant structural differences compared to the crystal structure of eVP40 dimer, the mVP40 dimer is found to adopt a configuration very similar to the eVP40 dimer after 200 ns of MD simulations. This conformational rearrangement upon lipid binding allows mVP40 to localize and stabilize at the membrane surface in a manner very similar to the eVP40 dimer. Once associated with the membrane, VP40 dimers assemble into higher oligomers and the oligomerization requires further large-scale conformational rearrangements that involve disengagement of some of the CTDs from the NTDs.^5,33^ While the slippery CTD–CTD interface is likely to be the main contributor to the filovirus flexibility,^5^ our results provide insight on how the flexibility of the dimer interface can also play an important role on the overall virion flexibility.

## Methods

The mVP40 dimer structure was taken from the X-ray crystal structure in the Protein Data Bank (PDB ID: 5B0V) and the missing residues were added with Modeller.^34^ The protein and plasma membrane systems (both with and without membrane) were built using the Charmm-Gui membrane Builder web interface.^35^ The plasma membrane consists of phosphatidylcholine (POPC), phosphatidylethanolamine (POPE), phosphatidylserine (POPS), palmitoylsphingomyelin (PSM), phosphoinositol (POPI) and cholesterol (CHOL) molecules. The distribution of different lipid molecules in the lower leaflet of the plasma membrane were in the number ratio of 20 : 11 : 33 : 18 : 9 : 7 (CHOL : POPC : POPE : POPS : POPI : PSM) representing the high complexity of the plasma membrane composition.^36,37^ For the mVP40-membrane system, the membrane consists of 284 lipids in the upper leaflet and 290 in the lower leaflet. Further detail of the PM composition used in the simulation is given in Table S1.† For comparison, a similar system was set up for the eVP40 dimer (PDB ID: ; 4LDB). The eVP40-membrane system consists of 147 lipids in the upper leaflet and 156 in the lower leaflet. Both systems were solvated with TIP3 water in cubic boxes and neutralized with 0.15 M KCl. The solvated system (protein, membrane, water and the neutralizing ions) contained a total of 231 567 atoms for mVP40 and 121 417 for eVP40.

All-atom molecular dynamics simulations were performed with the CHARMM36 force field^38^ using NAMD 2.11.^39^ The particle mesh Ewald (PME) method^40^ was used to calculate the long-range ionic interactions. The covalent bonds involving hydrogen atoms were constrained by SHAKE.^41^ For each system, a 10 000-step minimization followed by equilibration runs were performed. Equilibration steps for the membrane systems were as described in Table S2.† This was followed by the NVT (constant volume/temperature) production runs at 300 K using 2 fs time steps. The pressure was controlled using the Nose–Hoover Langevin-piston method,^42^ with a piston period of 50 fs and a decay of 25 fs. Similarly, the temperature was controlled using the Langevin temperature coupling with a friction coefficient of 1 ps^–1^. Visualization of the trajectories and rendering were done with VMD.^43^

## Results

**Fig. 1:**
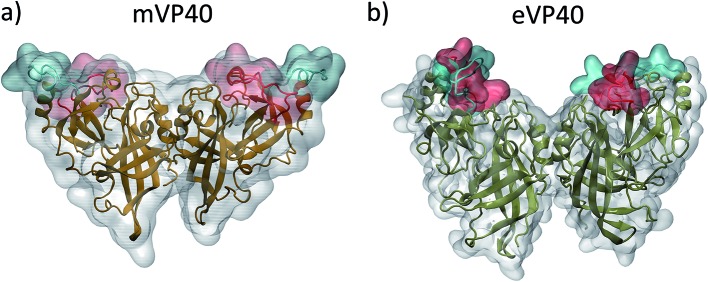
Structure of the (a) mVP40 dimer and (b) eVP40 dimer. The loop 1 residues are highlighted in blue and loop 2 residues are highlighted in red.

Comparison of the crystal structures of mVP40 and eVP40 dimers shows a significant structural difference in the membrane-interacting interface. As shown in Fig. 1, the CTDs on either side of the VP40 dimers contain a basic patch that interacts with the cytoplasmic leaflet of the plasma membrane. The basic patch residues reside in two different loop regions. In mVP40, loop 1 (residues 208–222) contains K210, K211, R215, and K218 and loop 2 (251–271) contains residues K259, K264, K265 and R266. Similarly in eVP40, the basic loop 1 (residues 219–233) contains K221, K224, K225 and loop 2 (residues 274–283) contains K274, K275, and K279. In Fig. 1, the basic loop 1 residues are highlighted in blue and the basic loop 2 residues are highlighted in red. The slightly lower positioning of the CTDs in mVP40 (Fig. 1a) allows a nearly flat and extended top surface that can interface with the membrane.^28^ This is in contrast to the crystal structure of the eVP40 dimer (Fig. 1b), which shows that the CTDs are positioned to give a chevron-like shape to the overall dimer with the NTDs at the bottom of the V-shape so that only the CTDs on either end can interface with the membrane. Comparison of the monomer–monomer interactions at the dimer interface in eVP40 and mVP40 shows that the eVP40 dimer interface is much more robust than the mVP40 interface. As shown in Fig. S1,† interfacial ionic/hydrogen bond interactions contribute significantly to the eVP40 dimer stability compared to the mVP40 dimer.

In order to investigate the membrane localization process of the mVP40 dimer in the plasma membrane, we initially placed the dimer slightly below the lower leaflet of the membrane, with all protein atoms located at >5 Å below any lipid atoms. It is known that the membrane localization of eVP40 requires POPS,^30^ so we included this lipid in the system. The strong electrostatic interactions between the negatively charged PS head groups in the membrane and the positively charged lysine residues in the CTDs allow the dimer to associate with the membrane. To understand the mVP40 dimer-membrane association and the resulting conformational changes, we performed a 300 ns all-atom MD simulation for the mVP40-membrane system described above.

### A. Plasma membrane association of the mVP40 dimer

**Fig. 2:**
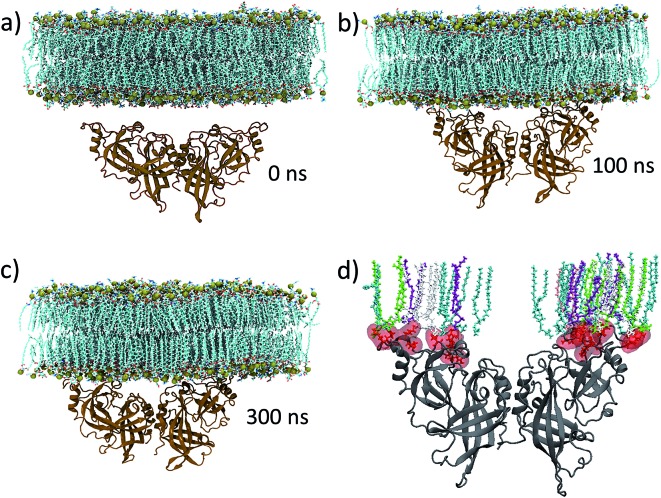
(a–c) Snapshots of the mVP40 dimer association with the plasma membrane at different times. (d) Various lipid types interacting with the basic loop 1 and basic loop 2 residues at 300 ns. The lipids are colored as: POPS-cyan, POPI-green, POPC-gray, POPE-purple.

We monitored the lipid–protein interactions during the association of the mVP40 dimer to the lower leaflet of the plasma membrane. Fig. 2a displays the initial configuration of the protein-membrane system after minimization and equilibration. Initially, the basic residues oriented towards the membrane were not too far away to interact strongly with the lipids. As the lysine residues in basic loops 1 and 2 start interacting with the anionic headgroups of lipids, the dimer gradually drifts towards the membrane, which is followed by further interactions with additional residues. By 50 ns, most of the basic patch residues have strongly interacted with the PS head groups as shown in Fig. 2b. In Fig. 2c, we show the final configuration of the mVP40 at the end of the 300 ns simulation. At this time, a significant number of lipids interact with the dimer (Fig. 2d).

**Fig. 3:**
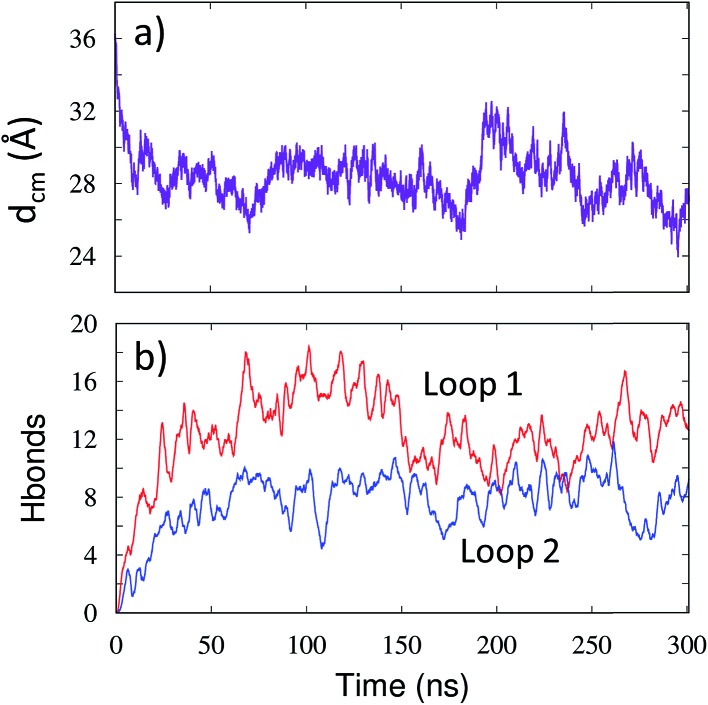
Time evolution of structural parameters during mVP40 dimer–membrane association. (a) Distance (along the *z*-axis) between the center of masses of the protein and the lipid bilayer (calculated using the center of mass of the phosphorous atoms). (b) The number of hydrogen bonds between the lipid head groups and the protein. The red and blue curves represent the hydrogen bonds formed with loops 1 and 2, respectively.

Fig. 3a shows the time evolution of the distance (*d*_cm_) along the *z*-axis between the center of mass of the protein and the lipid bilayer (calculated as the center of mass of the phosphorous atoms). The center of mass distance decreases gradually as the protein approaches the membrane. Movie S1† shows the overall drift of the mVP40 dimer towards the membrane and the resulting membrane association. After about 80 ns, a slight but interesting increase in *d*_cm_ is observed. This is caused by conformational changes in the mVP40 dimer as explained later.

Fig. 3b displays a plot of the hydrogen bonds for basic loops 1 and 2 and shows that the lipid interactions with the basic residues are important for the dimer's membrane localization and stability. The hydrogen bonds were calculated with 3.5 Å distance cut-off and 30° angle cut-off. A significant number of hydrogen bonds are formed between the protein and the lipid head groups. We observed that the number of hydrogen bonds for loop 1 is in general more than that for loop 2, showing a greater role of the loop 1 residues in membrane association and stabilization of the protein at the lower leaflet of the plasma membrane. Further details of the amino acids and lipids involved in the hydrogen bonding are discussed below.

### B. Lipid–protein interactions and lipid specificity

**Fig. 4:**
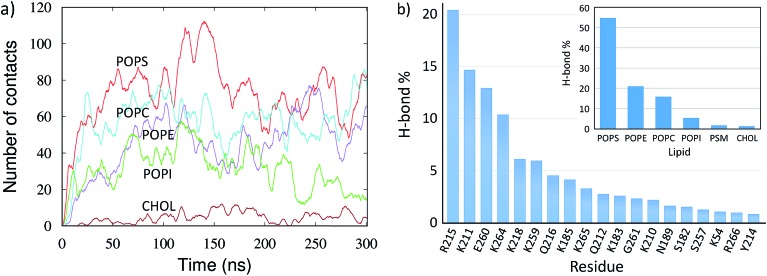
Lipid–protein interactions for the mVP40 dimer at the lower leaflet of the PM. (a) The number of contacts between the lipids and protein heavy atoms for various lipid types. (b) Relative percentage of the hydrogen bonds between the lipids and each interacting protein residues. Inset: relative percentage of the hydrogen bonds made with protein by each lipid type.

The mVP40 dimer association shows preferential lipid selectivity. As shown in Fig. 2d, almost all lipid types can be observed around the loop 1 and loop 2 regions. The electrostatic interactions with the basic residues are mostly made by POPS (colored cyan in Fig. 2d). We calculated the number of lipid–protein contacts for various lipid types and plotted the results in Fig. 4a as a function of time. The protein heavy atoms were considered to be in contact with lipid heavy atoms if they were within 3.5 Å of each other. As the dimer approaches the membrane, the number of contacts for all lipid types increases, with the exception of cholesterol, which is expected because the cholesterol head group is slightly buried inward compared to other lipid head groups in the bilayer. We find that mVP40 has more contacts with POPS than other lipid types. This agrees well with the experimental observation that POPS is important for plasma membrane localization for both eVP40 and mVP40.^29–31^ In addition to POPS, other lipids (POPE, POPC, and POPI) also have significant contacts with the protein atoms, mostly in the basic patch residues, which provide the electrostatic interactions for the mVP40 membrane association and stabilization at the lower leaflet.

We calculated the hydrogen bonds between the lipid and protein atoms for the last 100 ns of the trajectory (200 to 300 ns), with a distance cut-off of 3.5 Å and angle cut-off of 30° between the donor and acceptor heavy atoms. The mVP40 dimer is fully associated with the PM and structurally stable during this time. Multiple lipid molecules/types are found to interact with the basic patch residues (Fig. S3 and Table S3†). Details of the lipid–protein hydrogen bonds, including the information of the specific donor or acceptor atoms involved in hydrogen bonding, are given in Table S3.† We calculated the relative contribution of each of the hydrogen-bonding residues to the total lipid–protein hydrogen bonds. As shown in Fig. 4b, R215 is found to make the most hydrogen bonds with the lipids (20%). This is followed by K211 (15%), E260 (13%), and K264 (10%). Other major contributors in the lipid–protein hydrogen-bonding are K218, K259, Q216, K183, G261, and K210.

The electrostatic interactions between the cationic arginine and lysine residues and the anionic lipid head groups provide the major stability for the mVP40 dimer at the plasma membrane. However, it is interesting to note that the negatively charged E260 is the third largest contributor to the overall hydrogen-bonding. This is also the only negatively charged residue making hydrogen-bonds with the membrane. To understand exactly how the E260 side chain interacts with the lipids, we explored the hydrogen bonds around this residue. As displayed in Fig. S4,† multiple POPS and POPE can hydrogen bond with E260. Specifically, anionic oxygen of E260 is the acceptor and the serine amino group of POPS and the amine group of POPE are the donors. It is worth noting that the lipid head groups also make an extensive network of hydrogen bonds that shield the E260 anionic side chain from the negative phosphate head groups of lipids, akin to solvent screening of a charged group, and can affect the lateral fluidity of the membrane.^44^ Such lipid–protein interactions can also cause lipid clustering.^45^

In Fig. 4b (inset), we also display the contribution of each lipid type to the overall lipid–protein hydrogen bonding. The relative population of the lipid specific hydrogen bonds shows that POPS, which contains the negatively charged head group, provides the dominant contribution (55%) to the lipid–protein electrostatic interactions. This is followed by POPE (21%) and POPC (16%). POPI, PSM and CHOL participate in very little hydrogen bonding with protein.

### C. Conformational changes in mVP40 dimer

**Fig. 5:**
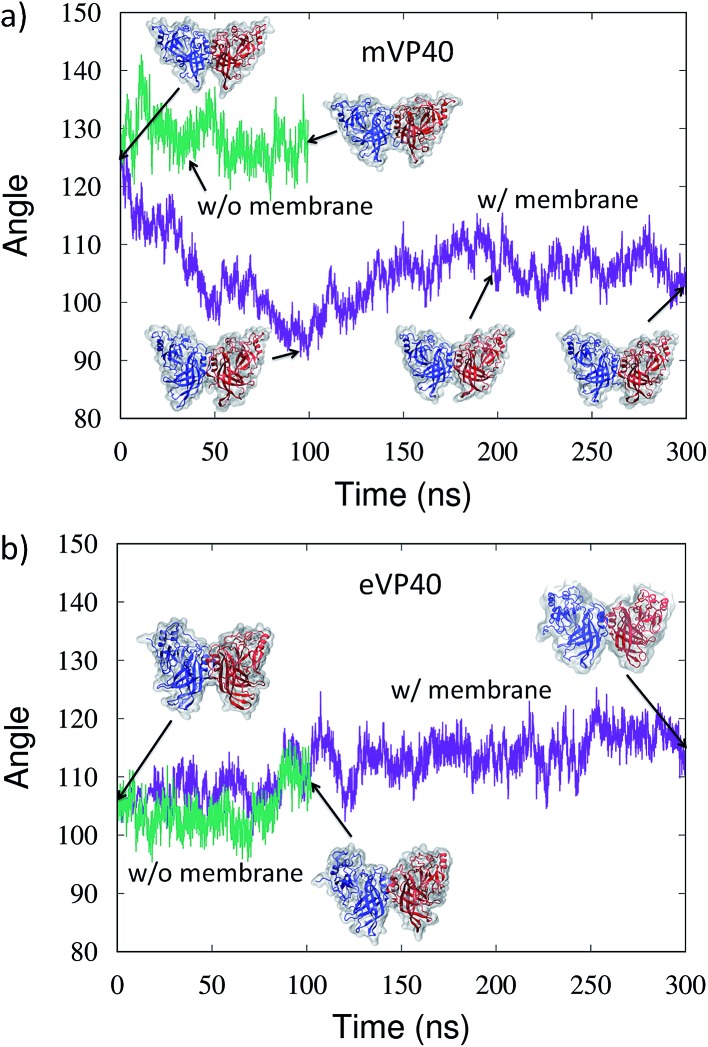
Time evolution of the angle (in degrees) between the monomers in the presence (purple) and absence (green) of lipid interactions for the (a) mVP40 dimer and (b) eVP40 dimer.

In order to determine any structural changes in the mVP40 dimer specifically due to membrane interactions, we compared various structural parameters for the mVP40 dimer simulated with or without membrane. Visualization of the mVP40-membrane trajectory clearly indicates that the two monomers in the dimer show a significant structural rearrangement due to lipid binding (ESI, Movies S2 and S3†). As the two CTDs at either end of the mVP40 dimer start interacting with the membrane, the relative orientations of the monomers start to change. The monomers can have twist, roll, and tilt motions about the monomer helices at the dimer interface. To monitor this conformational change, we calculated the angle between the best fit lines of the monomers and display the results in Fig. 5. For each monomer, both the CTD and NTD residues were considered for the angle calculation.

In Fig. 5, we plot the angle between the monomers for both mVP40 and eVP40 and compare how they evolve in the presence and absence of membrane. Initially, the angle between the monomers in mVP40 dimer is ∼20° more than in eVP40, with an angle of ∼125° for mVP40 and ∼105° for eVP40. This difference in the monomer relative orientations contributes to a flatter interface in mVP40 as shown in Fig. 1. As the basic residues in the CTDs of the mVP40 dimer interact with the membrane, the angle between the monomers gradually decreases due to reorientation of the monomers (Fig. 5a). This is in contrast to the behavior shown by eVP40 dimer. The angle between the monomers in eVP40 dimer starts out at a much lower angle but increases slightly with membrane interactions and settles to ∼115° (Fig. 5b). The decrease in the angle in mVP40 is quite significant, with as much as a 35° drop around 100 ns during the simulation. In fact, the angle in mVP40 is reduced to values lower than in eVP40 during the trajectory. Ultimately, the angles for both appear to converge to ∼110–115°. Still, these conformational changes in the mVP40 dimer as it associates with the membrane are more significant compared to those in the eVP40 dimer. To compare with the conformational changes in the absence of membrane interactions, we performed 100 ns all-atom simulations without the membrane as controls for both the mVP40 and eVP40 dimers. Except for the absence of a membrane, all other conditions were kept the same. As shown in Fig. 5a and b, both mVP40 and eVP40 dimers retain their overall shapes in the absence of the membrane interactions for the simulated timescales. The mVP40 without membrane curve (Fig. 5a) remains around 128°, significantly higher than the eVP40 without membrane value of 110°. This large difference in the conformation of the mVP40 dimer compared to the eVP40 dimer decreases when both associate with a membrane.

Inspection of the dimeric interface during the conformational change revealed the dynamic nature of the antiparallel β-sheet formed from residue segment 40–46 on each monomer. As mVP40 interacts with the membrane and the angle between the monomers starts to decrease, the hydrogen bonds between the β-strands rearrange slightly. Interestingly, a complete dissociation of the strands is observed after around 80 ns. The lowest angle observed at ∼100 ns is also marked by the largest separation between residues T40 and Y43, with no hydrogen bonds across the β-strands for about 20 ns. The β-strands then start to re-associate but with a slightly different H-bond pattern. The increase in the angle between monomers right around this time (120 ns) suggests a direct correlation between the monomer orientations and the β-strand dynamics at the interface. The dynamic motion of the β-strands is consistent with the experimental observations^32^ of significant deuterium incorporation at longer timescales, which suggested structural fluctuations at this region.

The conformational change in the mVP40 dimer also affects the lipid–protein interactions and therefore the flexibility of the residues at the membrane interface. As a measure of the residue flexibilities, we calculated the root-mean-squared fluctuations (rmsf) for all residues in the mVP40 dimer. Fig. S2† shows the rmsf values for all residues in the dimer at various time windows, calculated from the simulation of the protein-membrane system as well as the rmsf of the residues in the protein-only system. As the protein associates with the membrane, the flexibility of the residues starts to decrease in general. Compared to the protein-only system, a significant reduction in the rmsf values can be seen for most residues in the protein-membrane system at early time windows (*e.g.* 0–5 ns). Interestingly, the rmsf values are found to increase at later timescales (*e.g.* 95–100 ns), including for the residues in the basic loop regions that directly interact with the membrane. We hypothesize that this increase in the residue flexibility is due to the conformational changes induced by the lipid interactions. Specifically, the basic loop residues interacting with the membrane can show enhanced flexibility during monomer reorientations. Indeed, recent hydrogen-deuterium exchange experiments^32^ have found that the basic loop 1 showed reduced solvent accessibilities (reduced deuterium incorporation) after a 10 s incubation with PS containing liposomes, compared to that in the absence of PS containing liposomes. The basic loop 1 interactions with POPS likely caused the reduction in deuterium incorporations.^32^ This study also suggested the mVP40 membrane association was a dynamic process with continuous association and dissociation events taking place,^32,46^ which may lead to different relative flexibilities of basic loop 1 residues and varying levels of deuterium incorporation.

**Fig. 6:**
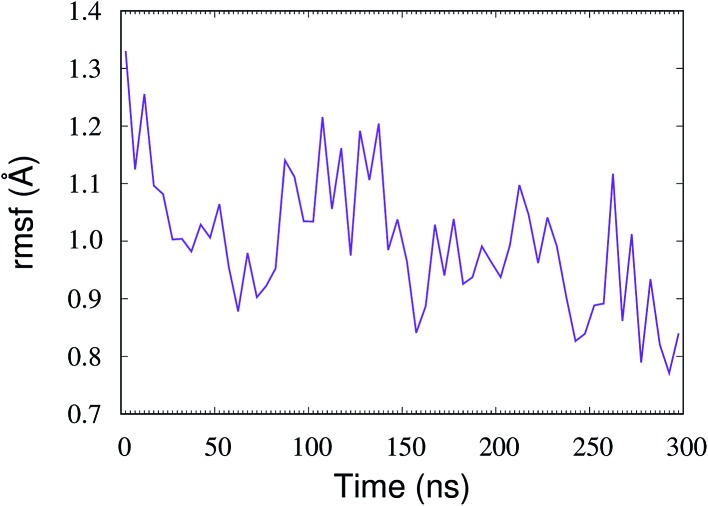
Time course of the flexibility of the basic patch residues (K210, K211, R215, K218, K259, K264, K265, and R266). For each point, the rmsf values of the basic loop residues were calculated for a time window of 5 ns, and averaged over the residues of the basic patch.

In order to understand the dynamic changes in the flexibility of the basic loop 1 due to the rearrangement of the mVP40 monomers, we calculated the rmsf values for every 5 ns window of the entire 0–300 ns trajectory, with a total of 60 windows. For each time window, we averaged the rmsf of the basic patch (K210, K211, R215, K218, K259, K264, K265, and R266) and plotted the average rmsf as a function of time in Fig. 6. The first data point in Fig. 6 at *t* = 0 was obtained from the 0–5 ns window of the protein-only system's trajectory and represents the flexibility of the basic patch in the absence of any lipid interactions. The time dependence of the average rmsf clearly shows a decrease in basic patch flexibility as the dimer associates with the membrane. By ∼80 ns, the average rmsf drops by more than 20%, after which it starts to increase slightly. As can be seen in Fig. 3a, the mVP40 dimer drifts steadily towards the membrane until ∼80 ns, evidenced by the decrease in the protein-membrane center of mass distance, *d*_cm_. During this time, the angle between the monomers also continues to decrease (Fig. 5). As discussed before, contacts between the β-stands (segments 40–46 from each monomer) dissociate and *d*_cm_ appears to reset its trend (Fig. 3a) to a slightly higher value after 80 ns, suggesting a reorganization in the protein structure. This is also marked by noticeable rearrangements of the lipid contacts shown in Fig. 4a between ∼80–160 ns. Therefore, structural reorganizations and the changes in the lipid contacts seem to result in an increased flexibility of the basic loop 1 during this time window (∼80–160 ns). As the new contacts are formed and loop 1 is stabilized (>160 ns), the loop flexibility is reduced again. These results provide more detailed insights on the dynamics of the mVP40 dimer at the PS containing membrane surface and help explain the deuterium exchange kinetics.

Although the membrane associated mVP40 dimer structure becomes more like the eVP40 dimer, some structural differences in the protein surfaces interfacing with the membrane still exist, which could lead to differences in the oligomerization and the budding of the VLPs. Indeed, recent studies examining lipid binding by eVP40 and mVP40 have found different degrees of anionic lipid selectivity for these two matrix proteins^29–31^ with eVP40 selectively associating with POPS and some phosphoinositides and mVP40 interacting with anionic lipids nonselectively based upon the anionic charge density of the membrane surface. Additionally, slight differences in eVP40 and mVP40 oligomerization may occur using a twisted hexameric filament at the plasma membrane interface for both filoviruses,^5,28^ but an alternative CTD oligomerization interface in the α4-helix has also recently been proposed for mVP40.^32^ The conformational flexibility of the NTD–NTD interface observed in mVP40 dimer may have significance in the viral budding and virion flexibility. In contrast, due to the rigidity of the NTD–NTD interface in the eVP40, it is thought that only the CTD–CTD interface between two hexamers can provide the flexible surfaces necessary for forming the flexible and pleomorphic filovirus virion.^5,47^

## Conclusion

In this work, we investigated the lipid–protein interactions as well as the lipid-induced conformational changes in the mVP40 dimer as it associates with the lower leaflet of the plasma membrane. We performed all-atom molecular dynamics simulations and identified important residues that facilitate the membrane association of mVP40 dimer and stabilize it at the PM. Results show that the hydrogen bonds between POPS lipid and residues K211, R215, and E260 dominate the overall lipid–protein interactions. We compared the structural changes of the mVP40 dimer with the eVP40 dimer in the presence and absence of membrane interactions. Despite the significant structural differences in the crystal structure, the mVP40 dimer is found to adopt a very similar configuration to the eVP40 dimer after associating with the membrane. As the two CTDs at the either end of the mVP40 dimer start interacting with the membrane, the relative orientation of the monomers that allows a nearly flatter top surface starts to change. Although the angle between the monomers in the mVP40 dimer is initially much wider than in the eVP40 dimer, the mVP40 angle decreases significantly due to lipid interactions. In contrast, the eVP40 dimer conformation does not show a significant change upon association with the membrane. Simulations of mVP40 and eVP40 in the absence of the membrane interactions showed that the dimers retain their overall different shapes, highlighting the role of the lipid interactions in facilitating the conformational changes in the mVP40 dimer. These conformational changes upon lipid binding allow mVP40 to localize and stabilize at the membrane surface similarly to the eVP40 dimer, but may give subtle differences in its function due to the differences in the solution conformations. In addition to providing a proper orientation for oligomerization into hexamers, the ability of mVP40 dimer to undergo conformational changes about the NTD–NTD interface may have significance in pleomorphic nature of the MARV virion. Finally, structural information in its lipid-interacting condition may prove useful in targeting mVP40 dimer for novel drugs to inhibit viral budding from the plasma membrane.
