# Supplementary material for: Plasma membrane association facilitates conformational changes in the Marburg virus protein VP40 dimer
Source: RSC Adv. 2017 Apr 26;7(37):22741–8. doi: 10.1039/c7ra02940c (PMC5436087; doi:10.1039/c7ra02940c)
Supplement: Supplementary file 4 [file RA-007-C7RA02940C-s004.pdf]

Electronic Supplementary Material (ESI) for RSC Advances.
This journal is © The Royal Society of Chemistry 2017

Plasma Membrane Association Facilitates Conformational Changes in the Marburg Virus Protein VP40 Dimer

Nisha Bhattarai[1], Jeevan B. GC[1], Bernard S. Gerstman[1,2], Robert V. Stahelin[3,4], Prem P. Chapagain[1,2*]

[1]Department of Physics, and [2]Biomolecular Sciences Institute, Florida International University, Miami, FL 33199, USA; [3]Department of Chemistry and Biochemistry, The Eck Institute for Global Health, and the Boler-Parseghian Center for Rare and Neglected Diseases, University of Notre Dame, Notre Dame, IN 46556, USA and [4]Department of Biochemistry and Molecular Biology, Indiana University School of Medicine-South Bend, South Bend, IN 46617, USA

*Email: chapagap@fiu.edu

Supporting Information

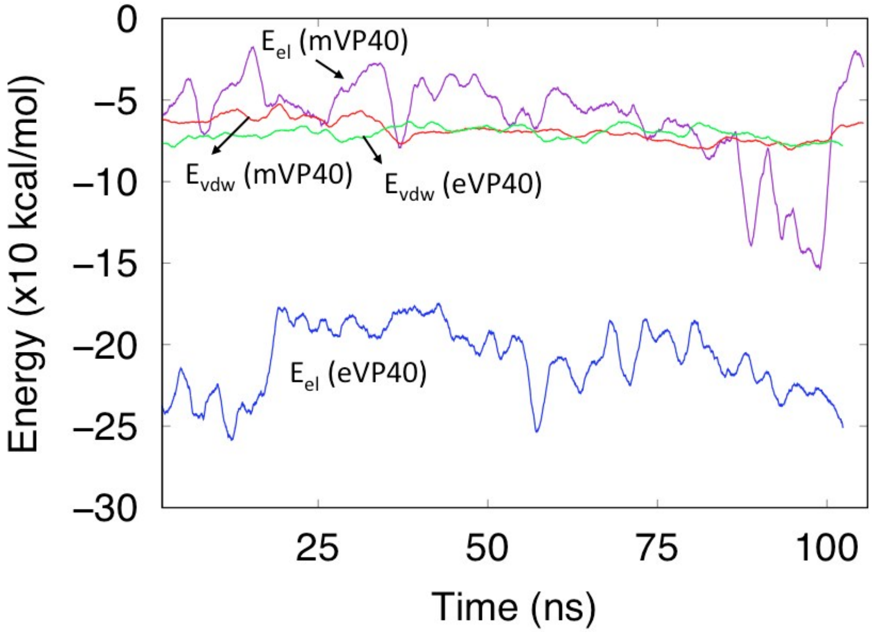

Fig. S1. Electrostatic (E_{el}) and van der Waals (E_{vdw}) energy between the monomers in both eVP40 and mVP40 dimers, calculated using NamdEnergy plugin in VMD. The dimer interface interactions in both eVP40 and mVP40 have similar van der Waals energy contribution but the interfacial electrostatic energy contribution is much larger in the eVP40 dimer interface compared to that in mVP40.

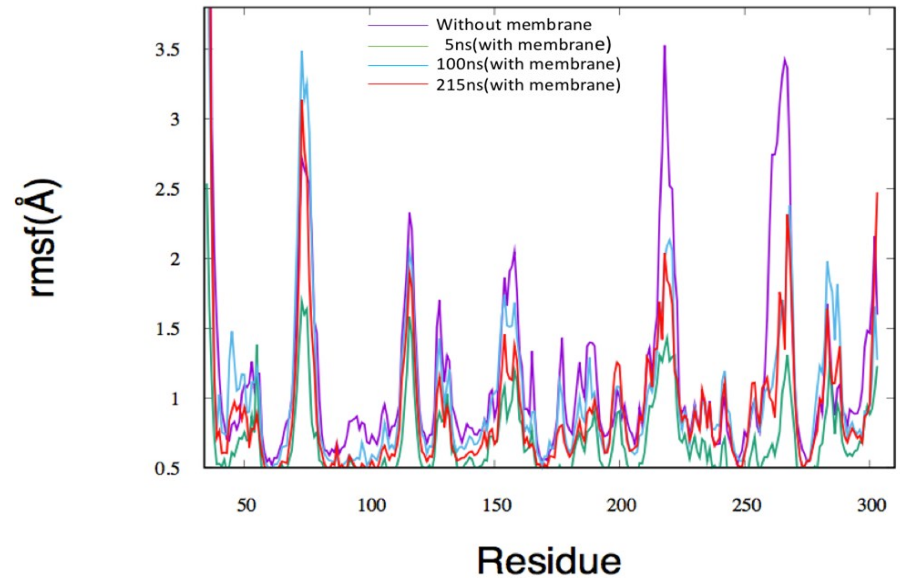

Fig. S2. Root-mean-squared fluctuations (rmsf) for all the residues in the mVP40 dimer at various time windows. As the protein associates with the membrane, the flexibility of all the residues decreases in general. The lipid interactions affect the CTD rmsf more than the NTD.

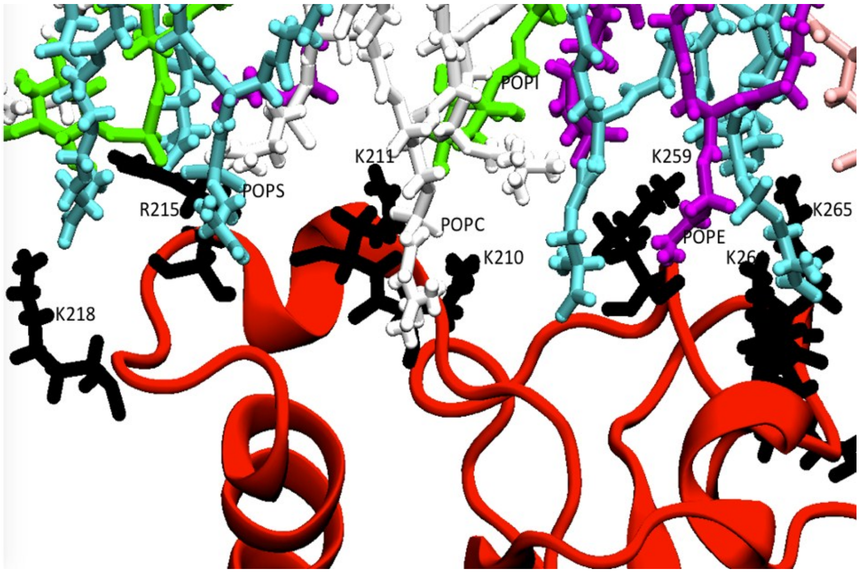

Fig. S3. Lipid-protein interactions between the basic patch residues in the mVP40-CTD and the plasma membrane lipids.

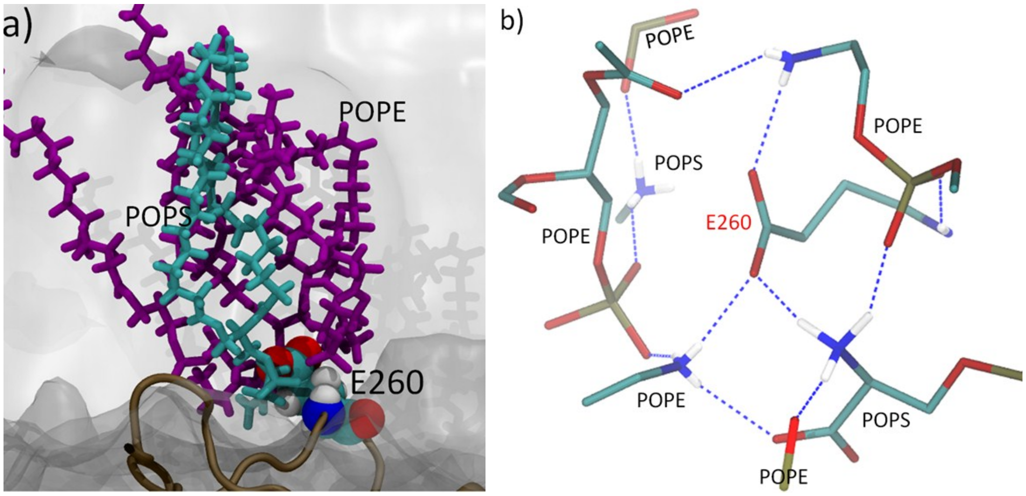

Fig S4: E260 interactions with the PM. a) Lipid molecules clustered around E260 (purple: POPE, cyan: POPS). Extensive network of hydrogen bonds formed between E260, POPE, and POPS.

Movie S1. A 300-ns simulation trajectory of the membrane association of mVP40. The lipid molecules interacting with the protein, as well as the interacting amino acids, are highlighted in VDW representation.

Movie S2. The conformational change of the dimer during the 300-ns of MD simulation (same trajectory as in Movie S1 but only the protein shown).

Movie S3. Animation of the mVP40 dimer conformational change obtained by interpolating the initial (0 ns) and final (300 ns) frames, using UCSF Chimera package (Pattersen et al, J. Comput. Chem. 25, 2004).

Table S1. Lipid bilayer setup

mVP40

Lipid type	*Upper leaflet*	*Lower leaflet*	*Lower Leaflet %*
CHOL	50	60	21
POPC	98	34	12
POPE	24	96	33
POPS	10	52	18
POPI	12	28	9
PSM	90	20	7
Total	284	290	100

eVP40

Lipid type	*Upper leaflet*	*Lower leaflet*	*Lower Leaflet %*
CHOL	28	32	21
POPC	48	17	11
POPE	12	48	32
POPS	5	25	17
POPI	6	14	9
PSM	50	15	10
Total	149	151	100

Table S2. Equilibration steps (for both eVP40 and mVP40 with membrane)

Steps	k(lipid head) kcal/(mol Å^2)	k(protein backbone) kcal/(mol Å^2)	k(protein side chains) kcal/(mol Å^2)	MD steps fs/timestep	Ensemble	Running time (ps)
1	5	10	5	1	NVT	25
2	5	5	2.5	1	NVT	25
3	2	2.5	1	1	NPT	25
4	1	1	0.5	1	NPT	100
5	0.2	0.5	0.1	2	NPT	100
6	0	0.1	0	2	NPT	100

Table S3. List of lipid-protein hydrogen bonds for the last 100 ns of simulation sorted by their relative occupancy (calculated by VMD with distance cutoff of 3.5 and angle cutoff of 30°). Only the hydrogen bonds with occupancy of >10% are listed.

**Donor**	**Acceptor**	**Occupancy**
ARG215-Side	POPS398-Side	153%
ARG215-Side	POPS366-Side	144%
POPS356-Main	GLU260-Side	91%
POPE416-Main	GLU260-Side	88%
GLN216-Main	POPC376-Side	80%
POPE444-Main	GLU260-Side	75%
LYS211-Main	POPC349-Side	73%
LYS211-Side	POPS398-Side	70%
LYS259-Side	POPE416-Side	69%
LYS211-Side	POPE400-Side	68%
LYS264-Side	POPS350-Side	62%
LYS211-Side	POPS374-Side	58%
LYS185-Side	POPS374-Side	56%
ARG215-Side	POPE412-Side	51%

LYS264-Side	POPS450-Side	50%
LYS218-Side	POPS364-Side	49%
ARG215-Side	POPS386-Side	47%
LYS211-Side	POPC349-Side	46%
GLY261-Main	POPS350-Side	43%
LYS185-Side	POPS353-Side	40%
ARG215-Side	POPS364-Side	38%
LYS259-Side	PSM389-Side	37%
LYS183-Side	POPS372-Side	37%
LYS210-Side	POPC349-Side	34%
POPS350-Main	GLU260-Side	34%
LYS264-Side	POPS378-Side	32%
SER257-Side	POPS356-Side	30%
ARG215-Side	POPC376-Side	28%
SER182-Side	POPS350-Side	20%
POPE358-Main	ASP184-Side	18%
LYS259-Side	POPC345-Side	17%
LYS265-Side	POPC361-Side	17%
GLN212-Side	CHL1342-Side	17%
POPI352-Side	GLN212-Main	16%
LYS265-Side	POPS378-Side	14%
LYS54-Side	POPS406-Side	14%
ASN189-Main	POPC351-Side	14%
TYR214-Side	POPS366-Side	14%
LYS265-Side	POPE441-Side	14%
LYS218-Side	POPS553-Side	14%
LYS183-Side	POPS370-Side	13%
LYS218-Side	POPS354-Side	13%
POPS350-Main	SER182-Side	13%
LYS264-Side	POPE427-Side	12%
LYS211-Side	POPE346-Side	12%
LYS210-Side	POPC359-Side	11%
GLU260-Main	POPE416-Side	11%
POPI396-Side	ASP184-Side	11%
LYS218-Side	POPE423-Side	10%
POPS450-Main	GLY261-Main	10%
ARG266-Side	POPI436-Side	10%
ASN189-Side	POPC351-Side	10%
ASN189-Side	POPC362-Side	10%
LYS264-Side	POPE451-Side	10%
GLN216-Side	POPC376-Side	10%
LYS218-Side	POPE433-Side	10%
